# Unexpected difficult airway due to an undiagnosed congenital lingual thyroglossal duct cyst in a neonate without stridor: A case report

**DOI:** 10.1002/ccr3.5475

**Published:** 2022-02-23

**Authors:** Yoshiki Kohashi, Tomohiro Yamamoto, Miki Igarashi, Hironobu Nishimaki

**Affiliations:** ^1^ Division of Anesthesiology Nagaoka Red Cross Hospital Nagaoka City Niigata Japan; ^2^ Division of Anesthesiology Niigata University Graduate School of Medical and Dental Sciences Niigata City Niigata Japan; ^3^ Division of Anesthesiology Niigata City General Hospital Niigata City Niigata Japan

**Keywords:** gastrointestinal symptoms, Hirschsprung's disease, lingual thyroglossal duct cysts, unexpected difficult airway, upper airway obstruction, video laryngoscope

## Abstract

Lingual thyroglossal duct cysts can be a rare cause of feeding difficulties in infants. Here, we describe a case of an infant with vomiting and feeding difficulty diagnosed with Hirschsprung's disease. However, she developed an unexpected difficult airway during anesthesia induction due to an undiagnosed lingual thyroglossal duct cyst.

## Graphical abstract

Lingual thyroglossal duct cysts can cause abdominal symptoms and difficult airway in infants, while they may be overlooked when stridor is not prominent. A detailed medical history and multifaceted examination are important when stridor worsens.

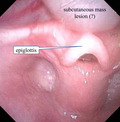

## 1 INTRODUCTION

Hirschsprung's disease is a common congenital anomaly that presents with abdominal symptoms such as abdominal distention, vomiting, constipation, and feeding difficulties shortly after birth.^1^ Congenital laryngeal cysts usually show symptoms associated with upper airway obstruction.^2^ However, it can be a rare cause of feeding difficulties in infants with individual differences in the onset time and the degree of symptoms,^2^, ^3^, ^4^ and can cause difficult airway; therefore, early and precise diagnosis is important. Here, we describe a case of an infant in whom an undiagnosed lingual thyroglossal duct cyst unexpectedly made ventilation and intubation difficult when securing the airway during anesthesia induction for surgery for Hirschsprung's disease.

Written informed consent for publication was obtained from the parents of the infant.

## 2 CASE HISTORY

Here, we describe a 28‐day‐old female infant (height, 51 cm; weight, 2.9 kg) who showed symptoms of poor weight gain due to feeding difficulties and post‐feeding vomiting. It was thought that Hirschsprung's disease was the cause of the gastrointestinal symptoms shortly after birth.^1^ She was scheduled to undergo a transanal Soave procedure^5^ for Hirschsprung's disease. No respiratory symptoms were identified in the preoperative examination. The tube feeding was stopped a day before the surgery. General anesthesia was slowly induced with sevoflurane and nitrous oxide. Through the established venous line, 15 mg thiamylal, 7 μg fentanyl, and 2 mg rocuronium were administered after confirming that assisted ventilation under spontaneous breathing was possible. However, mask ventilation became difficult, and the patient developed hypoxemia and bradycardia; her lowest heart rate was 70 per minute, and the saturation of peripheral oxygen (SpO_2_) could not be determined accurately. The anesthesiologist then requested the support of a senior anesthesiologist and a video laryngoscope (Pentax Airway Scope® [Pentax‐AWS®, Nihon Kohden]). Fortunately, ventilation improved and SpO_2_ recovered gradually after the senior doctor provided mask ventilation. However, endotracheal intubation was difficult because the glottis was not visible using a Macintosh curved‐blade laryngoscope (size 0). Successful intubation using an uncuffed tube with an inner diameter (ID) of 3.0 mm was achieved only on the sixth attempt by two anesthesiologists after changing a Macintosh curved‐blade laryngoscope (size 0) into a Miller straight‐blade laryngoscope (size 0) to lift the epiglottis before the video laryngoscope arrived. The surgery was performed as planned, and the patient was transferred to the neonatal intensive care unit (NICU). She was then extubated on postoperative day (POD) 2.

## 3 DIFFERENTIAL DIAGNOSIS

**FIGURE 1:**
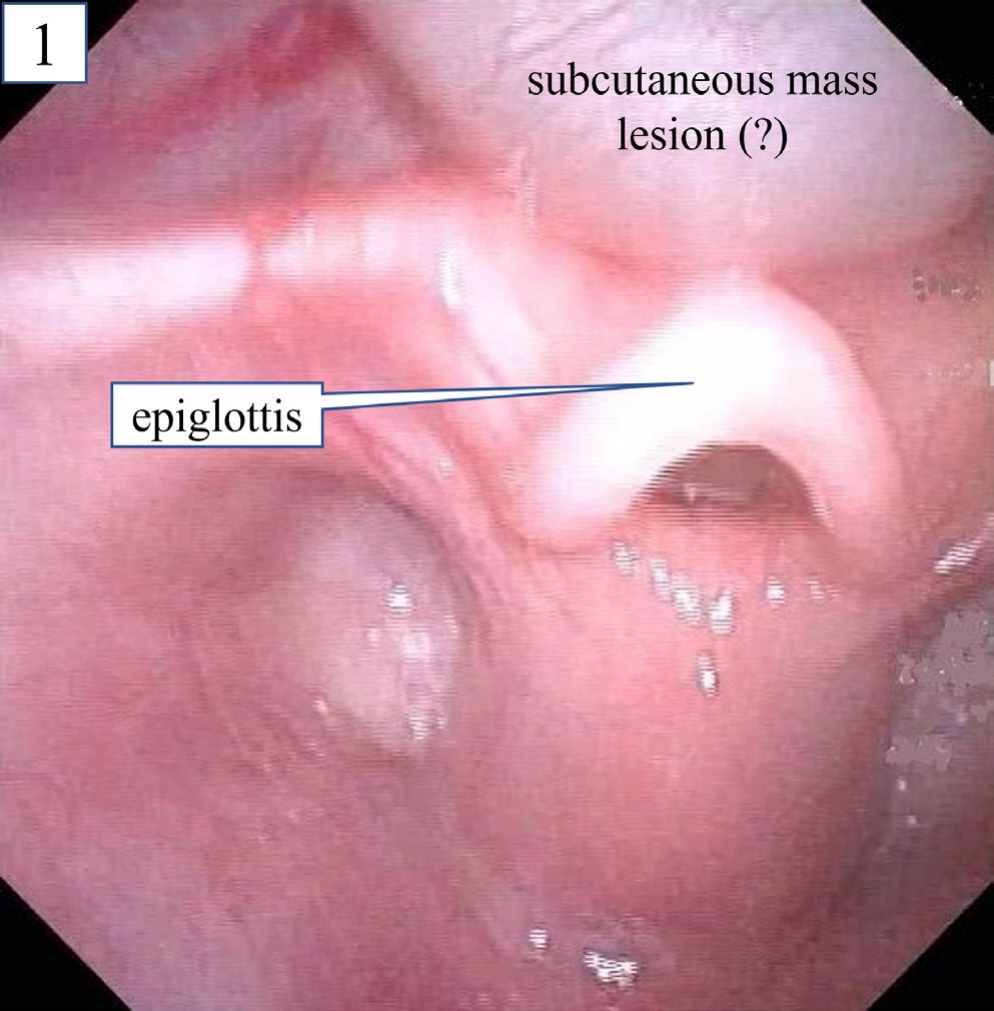
First laryngeal endoscopy on postoperative day 8. The findings suggested the presence of a subcutaneous mass lesion on the anterior side of the epiglottis; when reviewed later, however, it could not be pointed out

**FIGURE 2:**
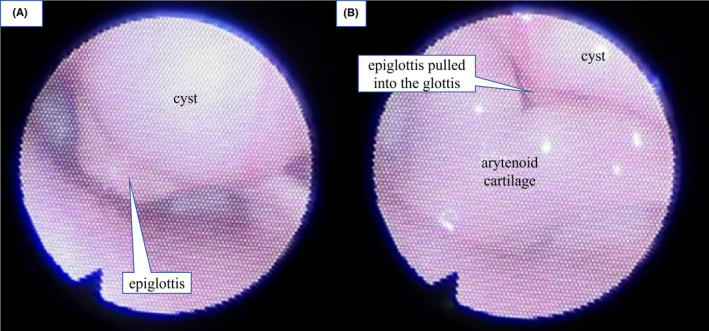
Second laryngeal endoscopy on postoperative day 21 revealed a large laryngeal tumor at the tongue base. (A) During exhalation, a large tumor at the tongue base obstructed the epiglottis from the anterior to the posterior side and the glottis was not visible. (B) During inspiration, the epiglottis was pulled into the glottis

**FIGURE 3:**
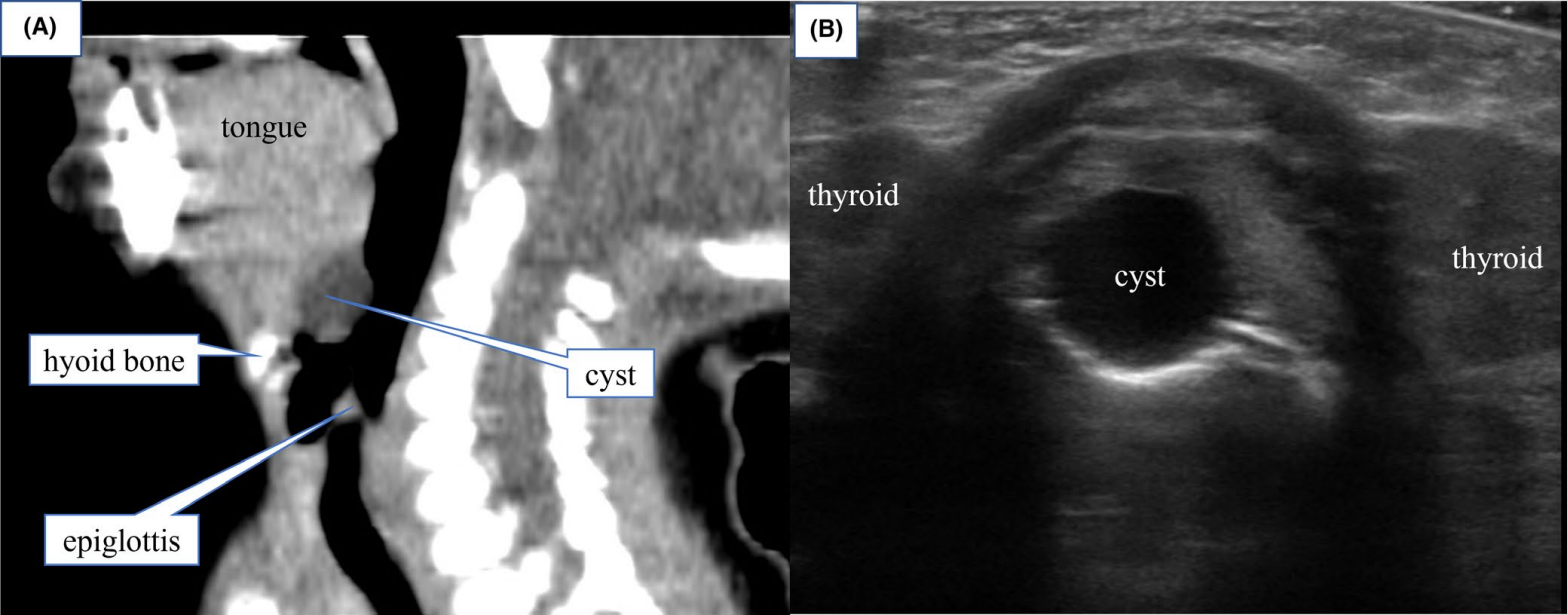
Computed tomography (CT) and cervical echography performed before laryngeal tumor resection. (A) Sagittal section of cervical CT showed a low‐density area about 1 cm in size at the tongue base. (B) Cervical echography revealed a cyst as a uniform hypoechoic region with a smooth margin

Despite the surgery for Hirschsprung's disease, the feeding difficulty continued, and prominent stridor developed. The first laryngeal endoscopy on POD 8 indicated laryngomalacia. Although, when later reviewed, a slight subcutaneous mass lesion on the anterior side of the epiglottis appeared, it was not pointed out at that time (Figure 1). The patient continued to have poor weight gain, and the inspiratory stridor during sleep worsened. Therefore, a second laryngeal endoscopy was performed on POD 21. A large laryngeal tumor that obstructed the epiglottis from the anterior to the posterior side was detected at the tongue base, with the epiglottis being pulled into the glottis during inspiration (Figure 2, Video S1). Computed tomography (CT) showed a cystic mass at the tongue base, and cervical ultrasonography also showed a cyst with smooth margins (Figure 3). This was determined to be a congenital laryngeal cyst.

**FIGURE 4:**
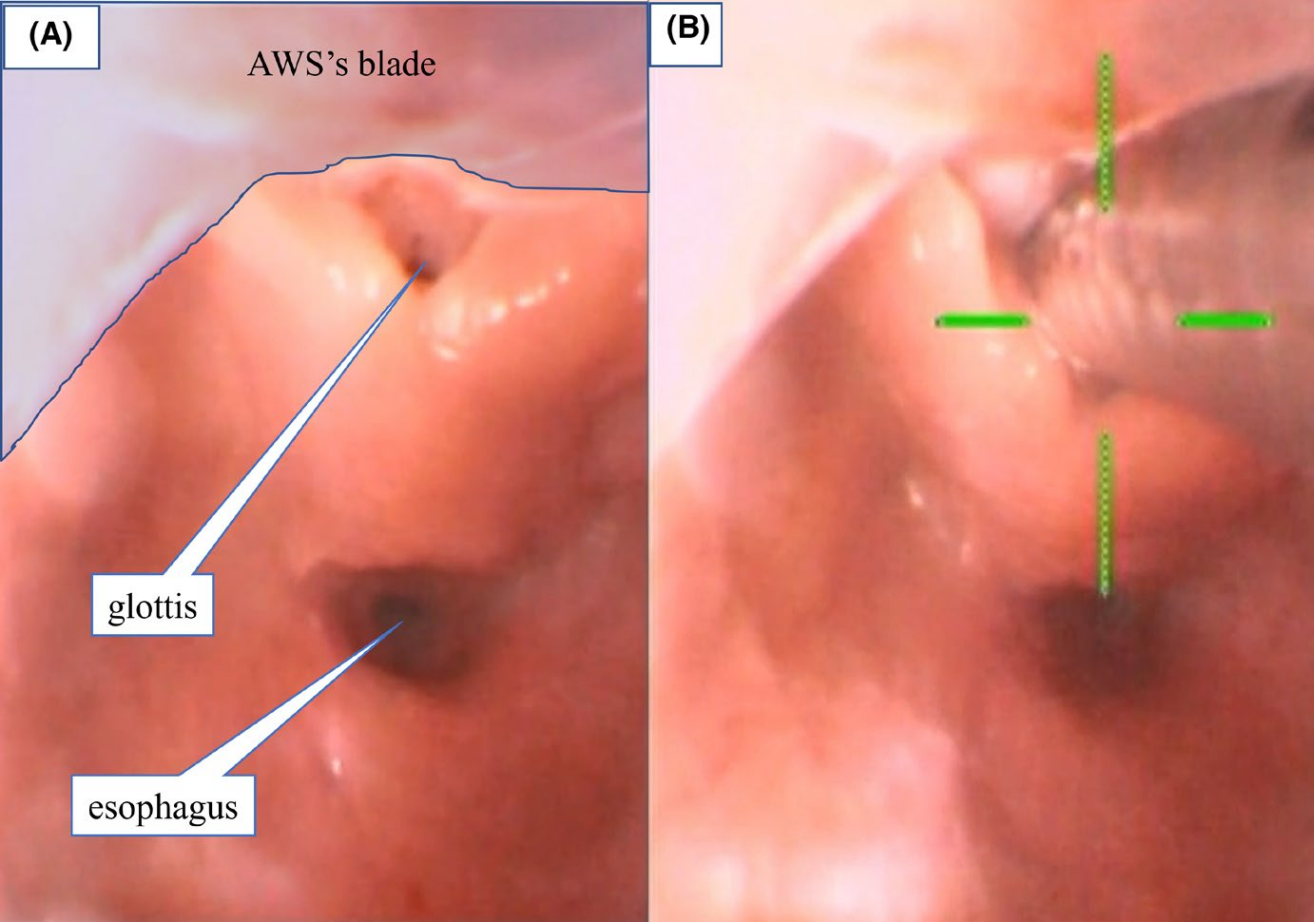
Image obtained using the video laryngoscope while securing the airway. (A) The glottis and esophagus were visible by lifting the epiglottis and the cyst upward. (B) Intubation with an uncuffed tube with an internal diameter of 3.5 mm was successfully performed using the video laryngoscope (Pentax‐AWS®)

Laryngeal cystectomy was scheduled on POD 27. Based on the published reports,^6^, ^7^ we planned to manage the airway with a video laryngoscope (Pentax‐AWS®). After establishing intravenous access in advance, slow induction was started with sevoflurane and nitrous oxide to preserve spontaneous ventilation, and assisted ventilation with a mask was possible. The glottis and esophagus were visible by lifting the epiglottis and the cyst upward with the video laryngoscope (Figure 4A). As the infant had large leaks with mechanical ventilation during surgery and postoperative NICU management of the first surgery using an uncuffed tube with an ID of 3.0 mm, a cuffed tube with an ID of 3.5 mm was prepared. We determined that intubation was possible because the tip of this tube could be inserted through the glottis, while the cuff would not pass. Therefore, 2 mg rocuronium and 8 μg fentanyl were administered. The intubation was successfully performed using an uncuffed tube with an ID of 3.5 mm (Figure 4B, Video S2). The cyst remained intact during the intubation manipulation. The cyst was completely resected by the otolaryngologist, as simple aspiration of the cyst is generally considered to have a high risk of recurrence.^8^ The postoperative chest X‐ray indicated no findings of aspiration. The patient was transported to the NICU and extubated three days after the laryngeal cystectomy. While her feeding difficulties gradually improved, mild stridor due to laryngomalacia remained. A thyroglossal duct cyst was diagnosed on the basis of histopathology. The cyst size was 21 mm × 10 mm × 7 mm, which was significantly larger than that seen on the CT. The patient was discharged 20 days after the laryngeal cystectomy.

## 4 DISCUSSION

Congenital laryngeal cysts are rare, with an incidence of 1.82 per 100,000 live births.^9^ Lingual thyroglossal duct cysts represent a more unusual variant that arises in the central tongue base.^10^, ^11^ The differential diagnosis includes vallecular cysts, dermoid cysts, adipose tumors, lymphangioma, hemangioma, and lingual thyroid.^4^ Most congenital laryngeal cysts have the potential to cause severe airway obstruction and sudden death or induce symptoms such as dyspnea, respiratory distress, and feeding difficulties within the first weeks of life.^3^ For neonates and infants, unlike adults, the symptoms of a lingual thyroglossal duct cyst are usually related to upper respiratory tract obstruction due to the limited space in the hypopharynx. However, these symptoms may sometimes be subtle and nonspecific, manifesting as dysphonia or mild feeding difficulties.^12^ Additionally, approximately 80%–90% of patients with Hirschsprung's disease typically develop constipation, abdominal distention, vomiting, and feeding difficulties and are diagnosed during the first few days of life.^1^ In the present case, the infant had only gastrointestinal symptoms before the first surgery for Hirschsprung's disease. She did not demonstrate typical symptoms of congenital laryngeal cysts such as inspiratory stridor. Therefore, it was not suspected that the feeding difficulties were due to anything other than Hirschsprung's disease.

Laryngeal endoscopy is important for diagnosing lingual thyroglossal duct cyst, but it may not always yield an accurate evaluation of the size of the cyst at the tongue base for crying or moving pediatric patients.^13^ Therefore, if infants have persistent stridor, evaluation by a pediatric otolaryngologist and cross‐sectional imaging with either CT or magnetic resonance imaging and cervical ultrasonography are indicated.^12^ If the test results in a suspected laryngeal cyst, an endoscopic examination should be performed again. In the present case, during the first surgery, the anesthesiologists attempted laryngoscopies several times but did not notice the cyst. When the mother was interviewed again in detail after the cyst was detected, she revealed a history of slight stridor during the infant's sleep before the first surgery for Hirschsprung's disease. Additionally, the symptoms of inspiratory stridor gradually worsened after the first surgery and the cyst was overlooked during the first laryngeal endoscopy after the operation on POD 8 (Figure 1). Therefore, we suspect that the cyst gradually increased in size, while its trigger is unknown, which could be due to external factors such as the repeated intubation attempts during the initial surgery, or due to inflammation, infection, and mucous retention naturally.^10^ Congenital laryngeal cysts should be considered as a possible differential diagnosis in patients whose stridor gradually worsens and is accompanied by feeding difficulties.

Airway management is the most problematic aspect of anesthesia in patients with congenital laryngeal cysts because of the risks of an obscured view of the larynx or cyst rupture during the intubation.^2^ Depending upon the size and location of lesions in the vallecular region in young pediatric patients, various methods such as rigid or flexible bronchoscopy, video laryngoscopy, light‐wand, retrograde intubation, and tracheostomy have been used to secure the airway.^14^ Fiberoptic intubation is considered one of the safest and most effective ways to secure a difficult airway. However, its use is associated with several limitations, such as minimal room for manipulation and glottis displacement due to the cyst.^15^ Moreover, infants quickly develop hypoxemia in a short period of apnea, and endotracheal tube insertion in younger pediatric patients also may be more difficult compared with older pediatric patients or adults despite an adequate view of the glottis.^16^ Considering these points and previous cases in which the Pentax‐AWS® enabled successful intubation,^17^ we used this device in the present case. This device allows for intubation to be performed quickly and for the epiglottis to be lifted to create space and provide a good field of view. Furthermore, its integrated tube‐insertion slot facilitates guidance of the endotracheal tube into the glottis. In addition, its blade is made of polycarbonate resin and airway complications such as bleeding from the pharynx with this device are lower compared with those seen when using a Macintosh laryngoscope.^18^

In conclusion, a lingual thyroglossal duct cyst is an unusual cause of vomiting and feeding difficulties. Therefore, it may be overlooked in Hirschsprung's disease in patients presenting with gastrointestinal symptoms without airway issues. The learning point of the present case is that lingual thyroglossal duct cysts can be a rare cause of feeding difficulties in infants and that congenital laryngeal cysts should be considered as a possible differential diagnosis in patients whose stridor gradually worsens and is accompanied by feeding difficulties. Therefore, a multifaceted examination is needed in patients whose stridor persists or worsens. In the present case, a video laryngoscope was an effective choice to manage the patient's airway, providing a good field of view to secure a difficult airway in an infant with a lingual thyroglossal duct cyst. The use of a video laryngoscope and supraglottic instruments should have been considered earlier in the first surgery.

## CONFLICT OF INTEREST

None.

## AUTHOR CONTRIBUTIONS

Yoshiki Kohashi performed perioperative anesthesia management, wrote the first draft of the manuscript, and prepared the figures and supplementary video. Tomohiro Yamamoto (Corresponding author) wrote the first draft of the manuscript and prepared the figures and supplementary video. Miki Igarashi helped to write the first draft of the manuscript. Hironobu Nishimaki assisted in the perioperative anesthesia management and helped to write the first draft of the manuscript.

## ETHICAL APPROVAL

None.

## CONSENT

Written informed consent for publication was obtained from the parents of the infant.

## Supporting information

Supplementary Material

Video S1

Video S2

## Data Availability

None.
