# Supplementary material for: Unexpected difficult airway due to an undiagnosed congenital lingual thyroglossal duct cyst in a neonate without stridor: A case report
Source: Clin Case Rep. 2022 Feb 23;10(2):e05475. doi: 10.1002/ccr3.5475 (PMC8864569; doi:10.1002/ccr3.5475)
Supplement: Supplementary file 1 — Supplementary Material [file CCR3-10-e05475-s002.docx]

**Supplementary Video Legends:**

**Supplementary Video 1: Laryngeal endoscopy on postoperative day 21 revealed a large laryngeal tumor at the tongue base.**

A large tumor obstructed the epiglottis from the anterior to posterior side at the tongue base, and the epiglottis was pulled into the glottis with inspiration.

**Supplementary Video 2: Successful intubation using a video laryngoscope.**

Intubation with an uncuffed tube with an internal diameter of 3.5 mm was successfully performed using a video laryngoscope (Pentax-AWS^®^).
